# Decomposing diversity into measures of evenness, similarity, and richness

**DOI:** 10.1002/ece3.10952

**Published:** 2024-02-13

**Authors:** Bingzhang Chen, Michael Grinfeld

**Affiliations:** ^1^ Department of Mathematics and Statistics University of Strathclyde Glasgow UK

**Keywords:** decomposition, diversity, evenness, Leinster–Cobbold diversity index, richness, similarity, taxonomic trees

## Abstract

It has long been recognized that diversity has many measurable aspects, such as richness, evenness, and similarity among species. However, given a diversity index, it is unclear whether it necessarily can be decomposed into components that reflect these different aspects. Here, we present a scheme to decompose the Leinster and Cobbold diversity index, which subsumes and generalizes many other indices, into the components of richness, evenness and taxonomic similarity. Our approach addresses the problem that in general a vector of equal relative abundances does not maximize diversity. Furthermore, our approach uses all available information to give unbiased estimates of both evenness and similarity.

## 1 INTRODUCTION

It has long been recognized that diversity has multiple aspects such as “richness” or “variety” reflecting the number of species present, dominance or rarity relations among the constituent species (“evenness” or “balance”), and “similarity” (or “disparity”) among the species (measures of distance between species based on taxonomic, phylogenetic, or functional traits relationships) (Daly et al., 2018; Leinster & Cobbold, 2012; Purvis & Hector, 2000; Stirling, 2007). Stirling (2007) even claims that “there seems no other obvious candidate for a fourth important general property of diversity beyond these three.”

In ecology, claimed biogeographical patterns of diversity often depend on the particular aspect of diversity being investigated. For example, Stuart‐Smith et al. (2013) show that biodiversity hot spots can shift from the tropics to higher latitudes if in addition to considering abundances, one takes account of functional traits similarity of species.

Several promising diversity indexes have been defined in the literature (Chao et al., 2014; Leinster & Cobbold, 2012; Rao, 1982; Stirling, 2007). However, a priori it is not clear whether from any given index one can extract information about richness, evenness, and species similarity. There is no argument why such a decomposition should exist and whether it has to be unique. In cases of non‐uniqueness, one has to define what is the optimal way to perform such a decomposition. The situation here is similar to the decomposition of γ diversity into α and β diversity indices either additively or multiplicatively (Anderson et al., 2011; Jost, 2007).

Being able to decompose a diversity index into biologically significant components would help us understand which aspect of diversity contributes to the changes in the relationship between diversity and environmental variables (e.g., latitude or productivity). Such a decomposition is also helpful in understanding how different aspects of biodiversity influence the functioning of ecosystems (Hillebrand et al., 2018; Maureaud et al., 2019).

In an important study, van Dam (2019) proposed a straightforward decomposition of the Leinster–Cobbold (2012) diversity index that contains information about the richness, evenness, and similarity aspects of diversity. However, as will be detailed below, van Dam's approach fails to consider the fact that a homogenous community (i.e., in which all constituent species have equal relative abundances) may not maximize diversity if similarity among species is taken into account. Leinster and Meckes (2016) showed that in general, there exists a unique abundance vector ***p*** * that maximizes diversity that can differ from the equal abundance vector which we denote by $p_{h}$. In other words, the maximal evenness of some communities is not achieved when all species have equal relative abundances. The criticism of van Dam's (2019) assumption that the component of evenness is maximal for an equal abundance vector can also be applied to the work of Daly et al. (2018) who stated that “the diversity measure is maximal for a fixed number of species *S* when all species' abundances are equal.”

In this study, we develop a decomposition approach that addresses the above problem. We focus on the Leinster–Cobbold (LC) index (Leinster & Cobbold, 2012) because it subsumes many other diversity indices that are widely used in ecology, such as the Hill numbers (Hill, 1973) and Rao's index (Rao, 1982; Ricotta & Moretti, 2011; Stirling, 2007), and has many other merits to be discussed later. For recent applications of this index, please see Mugabushaka et al. (2016) and Veresoglou et al. (2014). We take advantage of intrinsic properties of the LC index (e.g., the existence of the vector ***p*** *) to achieve an unbiased (in a sense to be explained below) decomposition of the index.

The structure of this article is as follows. In Section 2, we discuss the notions of richness, evenness, and similarity using the material in Chao et al. (2014), Chiu et al. (2014), Daly et al. (2018), and Gregorius and Gillet (2022). Then, in Section 3, we comment biodiversity indices in general and present the required information about the LC index following Leinster and Cobbold (2012) and Leinster and Meckes (2016). In Section 4, we present first van Dam's, and then our decomposition and its consequences. Finally, in Section 5, we discuss the merits and limitations of our approach and suggest future work.

We also note that even if the quotation from Stirling (2007) reflects a deep property of biodiversity indices, it is not clear that a decomposition of a biodiversity index cannot contain additional terms. In fact, one interpretation of the decomposition we suggest contains an additional term, related to Hill's numbers and to the maximal diversity of a biocommunity (defined below). That is, the additional term does not carry any new information but is needed “to balance the books.” The quotation from Stirling also does not prescribe how richness, evenness, and similarity should be defined.

## 2 DIVERSITY COMPONENTS

### 2.1 Notation

For clarity, we first establish notation. Everywhere below we assume that the number of species (i.e., richness) in a community is fixed at n>1.

We use $p=\left(p_{1},\ldots ,p_{n}\right)$ to denote the unit vector of relative abundances, that is,

$$p_{i}>0\;\text{for}\;\mathrm{all}\;i=1,\ldots n\;\text{and}\;\sum_{i=1}^{n}p_{i}=1.$$

We use the vector

(1) $$p_{h}=\left(\frac{1}{n},\ldots ,\frac{1}{n}\right),$$

where the subscript h stands for “homogeneous” to represent the equal relative abundance of species in a community. Below **1** will stand for an *n*‐vector with all components equal to 1.

Next, we need to discuss n×n matrices to record the information of species similarity. We denote the n×n identity matrix by $I_{n}$ which indicates that all species in the community are totally dissimilar with each other. We also use the notation $J_{n}$ for the n×n matrix of ones which represents an extreme case that all species are the same in the community (i.e., effectively we have only one species).

In this article, for simplicity, we will work with *ultrametric* matrices; this choice is motivated by the fact that similarity matrices (see Section 3.2) constructed using taxonomic trees are necessarily ultrametric and using ultrametric matrices simplifies the theory of Leinster and Mecke (2016). For more information on ultrametric matrices, please see Dellacheria et al. (2014), Leinster (2013), and Leinster and Meckes (2016).

### 2.2 Evenness

Classically, the “most even” population of *n* species is one for which the vector of relative abundances is the homogeneous vector $p_{h}$, that is, one where every species is equally represented (Daly et al., 2018). For thorough discussions of evenness see, for example, Chao et al. (2014), Chao and Ricotta (2019), Gregorius and Gillet (2022), and references therein. As the case of richness discussed below, the term “evenness” itself seems to be precluding discussion. As rightly pointed in Gregorius and Gillet (2022), a definition of maximal evenness in terms of $p_{h}$ leaves open the discussion of what would constitute “maximum unevenness.” Instead of “evenness,” van Dam (2019) uses “balance,” which seems to us a better term; this is the concept which, after defining it properly (see (12)) we will use below.

### 2.3 Richness

Richness is usually defined as the number of species (Daly et al., 2018). Such a definition is open to the same criticism as the notion of maximal evenness defined by $p_{h}$ discussed above. It is “species‐centric” without considering species similarity, and takes into account only the last level of taxonomic classification. We prefer to call the number of species *n* “species richness.”

### 2.4 Similarity

In this section, we discuss the construction of taxonomic similarity matrices *Z* for a community with *n* species.

**FIGURE 1:**
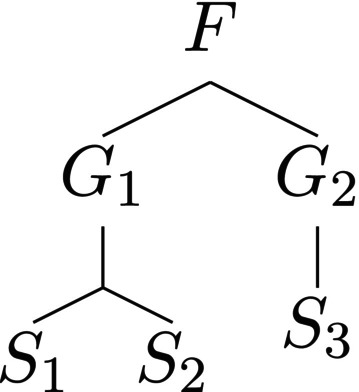
An example taxonomic tree with the root at the family level.

The usual way of constructing similarity matrices *Z* (which are automatically ultrametric) is to assign distances between different levels of a taxonomic tree. Then the taxonomic distance $d\left(i,j\right)$ between two species is the sum of distances from the nodes corresponding to these species to the first common node. Once the distance between nodes is defined, we could put $Z_{\mathrm{ij}}=e^{-d\left(i,j\right)}$ or, if the maximal distance in the tree has been normalized to 1, we could put $Z_{\mathrm{ij}}=1-d\left(i,j\right)$. As an example, consider the tree in Figure 1 and set the species–genus and the genus–family distance to be 0.3. If we use the additive recipe for defining *Z*, that is, put $Z_{\mathrm{ij}}=1-d\left(i,j\right)$, we obtain the similarity matrix

(2) $$Z_{1}=\left(\begin{array}{ccc} 1 & 0.7 & 0.4\\ 0.7 & 1 & 0.4\\ 0.4 & 0.4 & 1 \end{array}\right).$$

## 3 THE LEINSTER–COBBOLD DIVERSITY INDEX

The Leinster and Cobbold (LC) diversity index was introduced in Leinster and Cobbold (2012). The LC index is a far‐reaching generalization of Hill numbers, for discussions of which, see Chiu et al. (2014). For details on its properties, see Leinster and Cobbold (2012) and Leinster and Meckes (2016); here, we just collect the bare minimum in the framework of taxonomic (ultrametric) similarity matrices; ultrametricity is the blanket assumption on similarity matrices we make from now on.

Note that compared to the diversity indices proposed by Chao et al. (2014), the LC index has the flexibility to take into account taxonomic, phylogenetic, and functional diversity simultaneously, although the resulting similarity matrices will in general not be ultrametric. We leave this as a more general case for future study.

Before we start discussing the LC index, let us review the requirements that have to be satisfied by any diversity index.

### 3.1 Requirements for an diversity index

Leinster and Cobbold (2012, pp. 482–483) formulate conditions that any biodiversity index should satisfy; they number nine such conditions. The LC index satisfies all of them, that is, in brief, the LC index provides effective numbers, is modular, has the property of replication, is symmetric, is not changed if a species identical to one of those already present is added or by adding a new species of zero abundance, decreases if similarity between species increases, is larger if similarities between species are ignored, and always takes a value between 1 and the number of species *n*. Hence, the LC index is a legitimate starting point for the decomposition work we are interested in. We also note that while Leinster and Cobbold (2012) write that any index with sensitivity parameter *q* should be a monotone decreasing function of *q*, they do not add this criterion to the requirements on an index, as we do below.

### 3.2 Definition of the LC index

As in the definition of Hill numbers, below $q\in \left[0,,,\infty \right)$ is the sensitivity parameter, measuring the (un)importance given to rare species. Then for a community of *n* species with relative abundance vector ***p*** and similarity matrix *Z*, we have

Definition 1 The LC diversity of order *q* is

(3) $$F\left(Z,p,q\right)≔{\left(\sum_{i=1}^{n}p_{i}{\left(Zp^{T}\right)}_{i}^{q-1}\right)}^{1/\left(1-q\right)}.$$

Leinster and Cobbold (2012) show that the LC index indeed satisfies all the requirements imposed on a diversity index in Section 3.1.

Note that Leinster and Cobbold (2012) use a different notation, similar to the Hill number notation in the literature; they denote the right‐hand side of (3) by${\;}^{q}D^{Z}\left(p\right)$. We prefer the notation used here as it clearly shows functional dependencies and allows easy generalization, which we discuss briefly in Section 5. We collected the required properties of the LC index below and in Section 3.3.

The LC index has the following properties:

- $F\left(p,Z,q\right)$ is a monotone decreasing function of *q*;
- $F\left(p,Z,q\right)<F\left(p,I_{n},q\right)$ for all *q* if $Z\neq I_{n}$;
- $F\left(p_{h},I_{n},q\right)=n$ for all *q*;
- $F\left(p,J_{n},q\right)=1$ for all *q*.

For proofs of (a) and (b), see Leinster and Cobbold (2012); the other statements are immediate.

Following Leinster and Meckes (2016), we now discuss the concept of a *maximally balanced abundance vector* for a community of *n* species with an ultrametric similarity matrix *Z* which is the key starting point of our decomposition approach.

### 3.3 p*: A crucial property of the LC index

Leinster and Meckes (2016) prove that for each similarity matrix *Z*, there exists a unique abundance vector ***p*** * that maximizes the diversity index $F\left(Z,p,q\right)$ for every value of $q\in \left[0,,,\infty \right)$. We call ***p*** * the maximally balanced abundance vector (which replaces ***p***_h_ if taxonomic similarity is taken into account) and call *F*(*Z*, ***p*** *, *q*) (which is independent of *q*) the maximal diversity of a biocommunity with similarity matrix *Z*.

If Z is ultrametric, computing ***p*** * is a simple matter of solving a system of linear equations and normalizing:

(4) $$p_{i}^{*}=\frac{w_{i}}{\sum_{j=1}^{n}w_{j}}$$

where w solves the system of equations Zw=1, with 1 is a column vector of ones.

For the similarity matrix *Z*_1_ in (2), we have

$$p^{*}\approx \left(0.286,0.286,0.429\right).$$

which differs from $p_{h}=\left(0.333,0.333,0.333\right)$. We can check that when q=0, $F\left(Z,p^{*},q\right)=1.522$ which is greater than $F\left(Z,p_{h},q\right)=1.508$. For q=1,2, $F\left(Z,p^{*},q\right)$ remains unchanged, but $F\left(Z,p_{h},1\right)=1.504$ and $F\left(Z,p_{h},2\right)=1.500$.

Note that an alternative way of computing ***p*** * is provided in Leinster and Meckes (2016, lemma 6).

**FIGURE 2:**
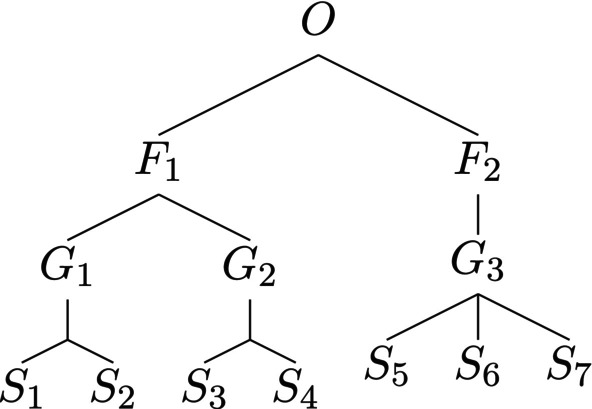
A taxonomic tree that does not have the same degree at all nodes but allows $p^{*}=p_{h}$.

There are similarity matrices $Z\neq I_{n}$ for which $p^{*}=p_{h}$. However, it is beyond the scope of this study to characterize exhaustively the set of such matrices. For now, we simply define a taxonomic tree to be *taxonomically equilibrated* if $p^{*}=p_{h}$. Of course if at each level of the tree all the nodes have the same degree, the taxonomic tree is equilibrated. However, the converse statement is not true. An example is provided by the tree in Figure 2.

**FIGURE 3:**
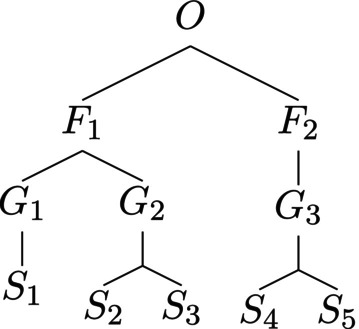
A taxonomic tree for which $p^{*}\neq p_{h}$ is guaranteed.

It is not hard to show that assigning of species–genus, genus–family, and family–order distances to be 0.25, and using the additive recipe to compute $Z_{\mathrm{ij}}$, results in a similarity matrix for which $p^{*}=p_{h}$. Thus, there is a trichotomy of taxonomic trees: those for which $p^{*}=p_{h}$ holds for every assignment of distances; those where such assignments can be chosen, as in Figure 2, and such that no assignment of distances results in a homogeneous maximally balanced abundance vector; an example of such a tree is shown in Figure 3.

## 4 AN UNBIASED DECOMPOSITION SCHEME

We are now ready to propose a decomposition scheme for the LC index. It is best to start with the decomposition scheme suggested by van Dam (2019) and see why it has to be modified. van Dam writes

(5) $$F\left(p,Z,q\right)=\frac{F\left(p,I_{n},q\right)}{F\left(p_{h},I_{n},q\right)}\cdot \frac{F\left(p,Z,q\right)}{F\left(p,I_{n},q\right)}\cdot F\left(p_{h},I_{n},q\right).$$

The second fraction in the right‐hand side of (5),

(6) $$D_{\mathrm{vD}}\left(p,Z,q\right)≔\frac{F\left(p,Z,q\right)}{F\left(p,I_{n},q\right)},$$

is clearly a measure of dissimilarity, while the first fraction should therefore be measure of balance,

(7) $$B_{\mathrm{vD}}\left(p,q\right)≔\frac{F\left(p,I_{n},q\right)}{F\left(p_{h},I_{n},q\right)};$$

$F\left(p_{h},I_{n},q\right)=n$, so the last term on the right‐hand side is the standard measure of richness. Note that, as it should be, the two fractions always lie in the interval $\left(1/n,1\right]$ (property (b) of the LC index ensures that the value 1/n cannot be reached).

The difficulty with (5) is in accepting (7) as a measure of balance. First of all, it does not use the information contained in the taxonomic similarity matrix Z. Secondly, it introduces $p_{h}$ which a priori has no meaning in theories that take similarity into account. $B_{\mathrm{vD}}\left(p,q\right)$ will take the maximal value 1 when $p=p_{h}$ for all values of q, while in the framework of the LC index, the measure of balance should be maximal when $p=p^{*}$. This argument makes the van Dam decomposition unusable in the framework of the LC index for which it is intended.

The dissimilarity measure $\frac{F\left(p,Z,q\right)}{F\left(p,I_{n},q\right)}$ does use information from both p and Z. Hence, the decomposition (5) can be said to be *asymmetrically biased*.

Note that an alternative decomposition

(8) $$F\left(p,Z,q\right)=\frac{F\left(p,Z,q\right)}{F\left(p_{h},Z,q\right)}\cdot \frac{F\left(p_{h},Z,q\right)}{F\left(p_{h},I_{n},q\right)}\cdot F\left(p_{h},I_{n},q\right),$$

in addition to having the drawbacks of (5) of using the often irrelevant abundance vector $p_{h}$ and having an asymmetrically biased dissimilarity measure, also has the problem that its measure of balance, the first term in the right‐hand side, can take values larger than 1 if Z is not a similarity matrix of an taxonomically equilibrated tree and $p=p^{*}$.

To ensure normalization and to be consistent with Leinster and Meckes (2016), consider the following decomposition:

(9) $$F\left(p,Z,q\right)=\frac{F\left(p,Z,q\right)}{F\left(p^{*},Z,q\right)}\cdot \frac{F\left(p^{*},Z,q\right)}{F\left(p^{*},I_{n},q\right)}\cdot F\left(p^{*},I_{n},q\right).$$

It is asymmetrically biased as the second factor, a dissimilarity measure, does not use the information from the actual abundance vector ***p***. We will discuss the interpretation of $F\left(p^{*},,,I_{n},,,q\right)$ later.

To obtain an **unbiased** decomposition, we multiply (5) and (9) and take a square root. The result is

(10) $$F\left(p,Z,q\right)=\sqrt{\frac{F\left(p,Z,q\right)}{F\left(p^{*},Z,q\right)}\frac{F\left(p,I_{n},q\right)}{F\left(p_{h},I_{n},q\right)}}\cdot \sqrt{\frac{F\left(p,Z,q\right)}{F\left(p,I_{n},q\right)}\frac{F\left(p^{*},Z,q\right)}{F\left(p^{*},I_{n},q\right)}}\cdot \sqrt{\mathrm{nF}\left(p^{*},I_{n},q\right)},$$

since $F\left(p_{h},I_{n},q\right)=n$.

Note that the last term in the right‐hand side of (10) can be rewritten as

(11) $$\sqrt{\mathrm{nF}\left(p^{*},I_{n},q\right)}=\sqrt{\frac{F\left(p^{*},I_{n},q\right)}{n}}n≔E\left(Z,q\right)n,$$

which is appropriate notation as $E\left(Z,q\right)$ is determined by Z. The term $E\left(Z,q\right)$ expresses the lack of equilibration in the taxonomic tree and always lies in the interval $\left(0,1\right]$. In the case of similarity matrices of taxonomically equilibrated trees, for which we have $p^{*}=p_{h}$ and hence $F\left(p^{*},I_{n},q\right)=n$, we have that $E\left(Z,q\right)=1$. If Z does not correspond to a taxonomically equilibrated taxonomic tree, $F\left(p^{*},I_{n},q\right)$ is dependent on q; $E\left(Z,q\right)$ is monotone decreasing in q; the limit of $E\left(Z,q\right)$ as q→∞ exists and may reflect properties of Z; we leave analysis of $E\left(Z,q\right)$ to future work.

The difficulty with the decomposition (10) as written is in interpreting

(12) $$B_{0}\left(p,Z,q\right)=\sqrt{\frac{F\left(p,Z,q\right)}{F\left(p^{*},Z,q\right)}\frac{F\left(p,I_{n},q\right)}{F\left(p_{h},I_{n},q\right)}}.$$

as a measure of balance as it is not necessarily maximized by $p=p^{*}$ for all values of q different from zero.

However, note that (10) can also be written as

(13) $$F\left(p,Z,q\right)=\sqrt{\frac{F\left(p,Z,q\right)}{F\left(p^{*},Z,q\right)}}\cdot \sqrt{\frac{F\left(p,Z,q\right)}{F\left(p,I_{n},q\right)}}\cdot \sqrt{F\left(p^{*},Z,q\right)F\left(p,I_{n},q\right)},$$

In both (13), we have an unbiased measure of balance,

(14) $$B\left(p,Z,q\right)≔\sqrt{\frac{F\left(p,Z,q\right)}{F\left(p^{*},Z,q\right)}},$$

which correctly takes its maximal value 1 at $p=p^{*}$, and an unbiased measure of dissimilarity

(15) $$D\left(p,Z,q\right)≔\sqrt{\frac{F\left(p,Z,q\right)}{F\left(p,I_{n},q\right)}},$$

which for any given ***p*** is maximized by choosing $I_{n}$.

We suggest to define

(16) $$R\left(p,Z,q\right)≔\sqrt{F\left(p^{*},Z,q\right)F\left(p,I_{n},q\right)},$$

the harmonic mean of Hill's number of order q, $F\left(p,I_{n},q\right)$, and $F\left(p^{*},Z,q\right)$, the maximal diversity for all q for a given similarity matrix Z (see Section 3.3), to be our notion of *effective richness* of order q. Note that it arises naturally in the process of unique unbiased decomposition of the LC index. This is not an inconsistent definition: if $Z=I_{n}$ and $p=p_{h}=p^{*}$, that is, for a maximally balanced population of totally unrelated species, we have that $R\left(p_{h},I_{n},q\right)=n$, the species richness, for all q≥0. So our notion of richness truly generalizes species richness and takes into account similarity among species, the abundance vector p and the sensitivity parameter q.

Hence, with this definition of richness, (13) becomes

(17) $$F\left(p,Z,q\right)=B\left(p,Z,q\right)D\left(p,Z,q\right)R\left(p,Z,q\right).$$

An advantage of our non‐“species‐centric” definition of richness is that it allows to compare two communities with different number of species.

## 5 DISCUSSION

We have proposed a decomposition of the LC index into components that include well‐defined measures of balance (evenness) and (dis)similarity. Compared to a previous version of decomposition (van Dam, 2019), our approach delivers measures of balance and of dissimilarity of the community that employ all the available information and do not use the homogeneous abundance vector $p_{h}$ that does not have any biological significance in realistic communities.

The price for having three components in the decomposition was having to redefine richness as in (16). We could alternatively write (13) as

(18) $$F\left(p,Z,q\right)=B\left(p,Z,q\right)D\left(p,Z,q\right)n\cdot \frac{R\left(p,Z,q\right)}{n},$$

in which case we must retain in our decomposition an in general *q*‐dependent fourth term.

However, if the choice is between (17) and (18), a decomposition into four terms, we argue that (17) is preferable: the last term in the right‐hand side of (18) can only be interpreted as the ratio between a measure of “effective richness” and the species richness and hence provides the same information as (17).

One could ask whether one could not start with the components (14), (15), n, and *define* an index $I\left(p,Z,q\right)$ by

$$I\left(p,Z,q\right)=B\left(p,Z,q\right)D\left(p,Z,q\right)n.$$

However, this “index” is not decreasing in *q*, so does not define a bona fide diversity index.

### 5.1 Incorporating more information streams

Although Stirling (2007) argues that there are only three aspects (i.e., richness, balance, and disparity) that should be considered in diversity and though LC index also uses only three “information streams”: the number of species n, the relative abundance vector p, and the similarity matrix Z, we could in theory consider a diversity index $F\left(c_{1},\;\ldots ,c_{m};q\right)$ with m>3, where $c_{1},\ldots ,\;c_{m}$ are various information streams. For example, one may wish to consider both the phylogenetic and functional diversity in the diversity index and we can have more than one Z. We could then follow the decomposition process of Section 4: find m! biased decompositions, multiply them together, take the m!‐th root, and simplify. However, this is already unwieldy in the case of m=3.

One approach to circumvent this problem is to take advantage of the flexibility of the LC index by incorporating different information streams into one unified similarity matrix. As explained in Section 2.4, one can define a similarity matrix by setting $Z_{\mathrm{ij}}=e^{-d\left(i,j\right)}$, where $d\left(i,j\right)$ is some suitably defined distance between species i and j. Hence, incorporating more information streams can be thought about as changing the distance function $d\left(\cdot ,\cdot \right)$. In the process of incorporating such information, such as functional similarity, the ultrametricity of the similarity matrix may be lost; it is possible that the resulting function $d\left(\cdot ,\cdot \right)$ will no longer be a metric, becoming more generally a divergence measure. The point is that p and (a suitably redefined) Z dependence of a diversity index is sufficient to incorporate all relevant information.

### 5.2 Limitations and future work

Our approach relies on the computation of ***p*** * which is not always possible if the similarity matrix Z is not ultrametric. Leinster and Meckes (2016) also note that if the number of species becomes high enough, the computation of ***p*** * in the non‐ultrametric case can be quite demanding. Extension of our work to the non‐ultrametric case relevant to phylogenetic trees and physiological traits similarity, and exploration of the ensuing computational issues is therefore a priority.

Sensitivity of similarity matrices, and hence the LC index and our decomposition, to the abundance of a species that is significantly distinct from other species in a community, is an area that needs to be explored.

Another good (linear algebra) research question is an exhaustive characterization of the trichotomy in taxonomic trees with respect to taxonomic tree equilibration, that is, understanding for which taxonomic trees any choice of similarity matrix leads to $p^{*}=p_{h}$, as opposed to ones only possible for particular choices of a similarity matrix, or impossible for any similarity matrix.

## AUTHOR CONTRIBUTIONS

**Bingzhang Chen:** Conceptualization (supporting); data curation (lead); funding acquisition (lead); investigation (supporting); methodology (supporting); software (equal); visualization (equal); writing – review and editing (supporting). **Michael Grinfeld:** Conceptualization (lead); formal analysis (lead); methodology (lead); writing – original draft (lead); writing – review and editing (lead).

## FUNDING INFORMATION

B. Chen was supported by a Leverhulme Trust Research Project Grant (RPG‐2020‐389).

## CONFLICT OF INTEREST STATEMENT

The authors declare no conflict of interest.

## Data Availability

This article does not involve any dataset.
